# 异基因造血干细胞移植患者巨细胞病毒感染管理中国专家共识（2022年版）

**DOI:** 10.3760/cma.j.issn.0253-2727.2022.08.001

**Published:** 2022-08

**Authors:** 中华医学会血液学分会干细胞应用学组

巨细胞病毒（cytomegalovirus, CMV）是一种属于疱疹病毒目乙型疱疹病毒亚科的双链DNA病毒，又称疱疹病毒5型，是导致异基因造血干细胞移植患者感染和预后不良的主要原因之一。近年来，随着单倍型和非亲缘造血干细胞移植的广泛应用以及CMV诊疗技术的发展，国外多个学术组织对造血干细胞移植后CMV感染的管理规范进行了更新。中华医学会血液学分会干细胞应用学组组织国内相关领域专家基于我国国情和循证医学证据，参考欧洲白血病感染会议和美国移植和细胞治疗学会等相关指南[1]–[4]制定我国第一版异基因造血干细胞移植患者CMV感染管理的专家共识。

一、CMV感染相关定义[5]–[6]

CMV感染定义为患者体液或组织中分离出病毒或检出病毒蛋白/核酸，包括以下5种类型：

1. 原发CMV感染：首次发生的CMV感染。

2. CMV激活：内源性潜伏CMV所导致的感染。

3. CMV病毒血症：在血标本中分离出病毒或检出病毒蛋白/核酸。包括：①CMV-DNA血症：在血标本中检测到病毒DNA；②CMV抗原血症：在外周血白细胞中检测到病毒PP65抗原。

4. CMV病：有临床症状和体征的CMV感染。包括：①CMV综合征：在CMV血症的基础上出现发热、乏力、骨髓抑制、转氨酶升高等非特异性症状，并排除其他原因引起的发热且无CMV终末器官疾病；②CMV终末器官疾病：CMV侵犯组织器官并导致相应症状和体征（如CMV肺炎、CMV胃肠炎）。

5. 难治性CMV感染：①难治性CMV病毒血症：接受合理的抗CMV治疗2周后，CMV病毒载量维持不变或上升。②难治性CMV病：接受合理的抗CMV治疗2周后，CMV病的症状、体征无改善或持续进展。

二、CMV感染的流行病学与危险因素

根据移植类型的差异，CMV感染的发生率为18％～85％，中位起病时间为移植后32～41 d[7]–[14]。亲缘全相合移植患者CMV感染的发生率为18％～55％[11]–[12],[15]–[16]，非亲缘相合移植为65％～70％[16]–[17]，脐血造血干细胞移植可达70％～85％[18]–[19]。单倍型移植在我国以抗胸腺细胞球蛋白（ATG）方案为主，CMV感染的发生率为60％～83％，中位起病时间为移植后32～37 d[11]–[13],[16],[20]–[21]。近期研究显示，ATG方案和欧美国家常用的移植后环磷酰胺（PT-Cy）方案CMV感染的发生率差异并无统计学意义[22]。

CMV可导致CMV病、急/慢性移植物抗宿主病（GVHD）、机会感染、骨髓抑制等多种不良事件，严重影响移植患者预后[20],[23]–[24]。其中CMV病在临床上最受关注。既往文献显示，CMV病的发生率为10％～40％，最主要类型为病死率高达70％的CMV肺炎[1]。目前由于预防和抢先治疗的广泛应用，CMV病的发生率已降至10％以下，但病死率仍高达20％～60％[1],[25]–[28]。

综合国内外文献报道，CMV感染高危因素包括年龄≥40岁、CMV IgG阳性、非同胞相合移植（脐血造血干细胞移植、单倍型移植、非亲缘移植）、GVHD预防方案（PT-Cy方案、大剂量ATG或阿伦单抗）、发生Ⅱ度以上急性GVHD及应用大剂量糖皮质激素（泼尼松≥1 mg·kg^−1^·d^−1^）等[2],[7]–[8],[27],[29]。

三、CMV感染的诊断

（一）CMV检测

1. 实时定量PCR（RT-qPCR）检测CMV-DNA：RT-qPCR被广泛应用于CMV感染的监测与诊断，其中血标本RT-qPCR因其高敏感性，成为CMV病毒血症首选的诊断技术[30]。但RT-qPCR也存在一些局限性，如检测方法尚未统一，不同检测技术的结果具有较大差异。建议各实验室采用WHO标准[31]并将结果报告为“每毫升国际标准单位（IU/ml）”。

2. CMV-PP65抗原检测：CMV-PP65抗原检测主要用于CMV血症的诊断，其检测结果与血标本RT-qPCR有着良好的一致性，但敏感性差于RT-qPCR[32]–[33]。CMV-PP65检测对于标本的采集、制备和检测人员操作熟练程度要求较高，目前不推荐作为CMV血症的首选诊断方法。

3. 病毒分离培养：传统的CMV培养技术费时费力，往往需要数周才能获得检测报告，因此不适用于临床诊断。快速培养技术可通过识别CMV即刻早期抗原而在24 h内得出结论，但是诊断的可靠性高度依赖操作者的熟练程度，敏感性也差于RT-qPCR和CMV-PP65抗原检测[34]–[35]。

4. 组织病理学检查：组织病理学具有高度的特异性，可作为CMV终末器官疾病诊断的金标准[34]–[35]。具体的方法包括传统的苏木精-伊红染色法和免疫组织化学分析。前者可在组织标本中发现细胞核和细胞质的包涵体，呈现典型的“鹰眼征”，后者通过检测组织中的CMV蛋白或核酸进行诊断。

（二）CMV病

CMV病多表现为非特异性临床症状，诊断主要依赖微生物学证据。目前国际上对CMV病采用“确诊”、“临床诊断”、“拟诊”的分级诊断（表1）[5]。本共识仅对常见的肺炎、胃肠炎以及视网膜炎进行论述。CMV病诊断标准详见表2。

**表1:** 巨细胞病毒（CMV）病分级诊断[5]

疾病类型	确诊	临床诊断	拟诊
CMV肺炎	是	是	是
CMV胃肠道炎	是	是	是
CMV视网膜炎	是	否	否
CMV脑炎	是	是	否
CMV肝炎、肾炎、心肌炎、胰腺炎、膀胱炎及其他终末器官疾病	是	否	否
CMV综合征	否	是	否

**表2:** 巨细胞病毒（CMV）病的诊断[5]

疾病类型	诊断级别	临床表现	微生物学证据
CMV肺炎	确诊	肺炎的临床症状和（或）体征，如影像学新发浸润、缺氧、呼吸急促和（或）呼吸困难	通过分离病毒、快速培养、组织病理学、免疫组织化学或DNA杂交技术在肺组织中发现CMV
	临床诊断	肺炎的临床症状和（或）体征，如影像学新发浸润、缺氧、呼吸急促和（或）呼吸困难	通过分离病毒、快速培养、定量CMV-DNA在支气管肺泡灌洗液中发现CMV
	拟诊	肺炎的临床症状和（或）体征，如影像学新发浸润、缺氧、呼吸急促和（或）呼吸困难	肺组织定量PCR检测发现CMV-DNA
CMV胃肠炎	确诊	①上消化道或下消化道的症状或体征；②内镜下发现黏膜损伤	通过组织病理学、病毒分离、快速培养、免疫组织化学或DNA杂交技术在局部组织中发现CMV
	临床诊断	上消化道或下消化道的症状或体征	通过组织病理学、病毒分离、快速培养、免疫组织化学或DNA杂交技术在局部组织中发现CMV
	拟诊	上消化道或下消化道的症状或体征	组织定量PCR检测发现CMV-DNA
CMV视网膜炎	确诊	经眼科专家判定具有典型的症状和体征即可确诊，如无法进行眼科检查或临床表现不典型，可根据核酸检测（如PCR）在玻璃体液发现CMV进行确诊	
CMV脑炎	确诊	中枢神经系统症状和体征	通过病毒分离、快速培养、免疫组化分析、原位杂交或定量PCR检测（最好）在组织中发现CMV
	临床诊断	①中枢神经系统症状和体征；②异常的影像学改变/脑炎脑电图改变	脑脊液发现CMV
CMV肝炎、肾炎、心肌炎、胰腺炎、膀胱炎及其他终末器官疾病	确诊	累及器官的相关症状和体征	通过病毒分离、快速培养、免疫组织化学分析或原位杂交在活检标本中检测到CMV，同时伴有CMV感染的组织学特征
CMV综合征	临床诊断	①发热≥38 °C至少2 d ②新发或加重的2度不适（CTCAE 4.0），新发或加重的3度疲劳（CTCAE 4.0） ③间隔24 h连续2次检测均显示白细胞减少或中性粒细胞减少。白细胞减少定义：出现症状前WBC≥4×10^9^/L的患者，WBC<3.5×10^9^/L；出现症状前WBC<4×10^9^/L的患者，WBC降幅>20%。中性粒细胞减少定义：中性粒细胞绝对计数（ANC）<1.5×10^9^/L；出现症状前ANC<1.5×10^9^/L的患者，ANC降幅>20% ④非典型淋巴细胞≥5% ⑤血小板减少：出现症状前PLT≥115×10^9^/L的患者，PLT<100×10^9^/L；出现症状前PLT<115×10^9^/L的患者，PLT降幅>20% ⑥肝转氨酶（丙氨酸氨基转氨酶或天冬氨酸氨基转移酶）上升至正常上限2倍（适用于非肝移植）	

1. CMV肺炎：CMV肺炎是最常见的CMV病，临床症状包括发热、咳嗽、气促、咯痰等非特异性表现，个别患者初期无症状，病情可迅速进展为急性呼吸衰竭。CMV肺炎影像学表现多样，“小叶中心结节”、“弥漫磨玻璃影”等间质性肺炎改变是CMV肺炎最主要的影像学特点[36]。支气管肺泡灌洗液CMV-DNA检测诊断阴性预测值可达100％，但对于支气管肺泡灌洗液CMV-DNA载量诊断折点仍有争议[1]。

2. CMV胃肠炎：CMV胃肠炎可发生于消化道的任何部位，但主要累及结肠，临床症状包括腹痛、发热、腹泻、便血等[37]。内镜下黏膜病损表现多样，“深大溃疡”是CMV结肠炎典型镜下特征[38]。血和粪便标本RT-qPCR对CMV胃肠炎的诊断价值仍有争议[26],[39]–[41]。初步研究显示，RT-qPCR检测新鲜组织标本CMV-DNA对诊断的阳性预测值、阴性预测值分别为93％、100％[42]。

3. CMV视网膜炎：CMV视网膜炎的临床表现包括飞蚊征、视物模糊、视力下降、畏光、视野缺损等，诊断主要依赖眼底镜检查，如发现出血和黄白色坏死灶混杂而成的典型“番茄炒鸡蛋”样表现，即可确诊[43]。如无条件进行眼底镜检查，也可以根据玻璃体抽取液检出CMV-DNA进行确诊[5]。

四、CMV监测

（一）移植前CMV血清学监测

供者与患者CMV血清学状态是影响移植后CMV感染的高危因素之一，也是供者选择的重要参考指标。我国人群中血清CMV IgG阳性比例大于90％，提示CMV潜伏比例高，因此供、患者IgG均为阳性的情况常见。供者IgG阴性患者IgG阳性的组合在中国人群中比例较低，但仍有发生，虽不影响CMV病的发生率，但会导致难治性CMV感染的比例增加[44]。

（二）移植后CMV病毒血症的监测

血标本RT-qPCR是移植后CMV病毒血症监测的首选方法。由于90％以上初次CMV病毒血症发生于移植后100 d内[27]，因此在这一时期应至少每周检测1次CMV血症。高危人群在移植100 d后仍有较高的CMV病毒血症风险，其发生率可达36％～54％，因此建议在该类人群适当延长监测时长、缩短检测间隔[1]–[2],[27],[45]–[46]。

建议：①移植前检测供、患者的CMV IgG水平以指导供者选择，IgG阳性患者应尽可能选择IgG阳性供者，IgG阴性患者应尽可能选择IgG阴性供者；②RT-qPCR作为CMV病毒血症首选的诊断方法；③移植后每周进行1～2次CMV病毒血症检测，持续至移植后100 d，对于高危人群建议适当延长监测时长、缩短检测间隔。

五、预防

（一）原发性CMV感染的预防

对于CMV IgG阴性患者，为了避免发生输血传播的原发性CMV感染，建议输注CMV血清学阴性或去白细胞血制品[47]。

（二）CMV激活预防

CMV激活预防是指CMV IgG阳性患者在诊断CMV病毒血症前应用抗CMV药物以防止CMV激活。更昔洛韦曾被用于移植后CMV预防，但因其严重骨髓抑制，目前临床应用较少。近期发表的随机对照研究显示，移植后28 d内至100 d使用来特莫韦进行预防能有效减少CMV激活，因此来特莫韦也获得国际指南的推荐[48]。但从性价比考虑，国外有中心将来特莫韦用于高危患者（单倍型移植、脐血造血干细胞移植、接受ATG预处理等）的CMV激活预防[2]。我国目前尚无来特莫韦用于预防的报道，相关研究有待开展。

（三）预防的启动时机和疗程

国际指南建议CMV激活预防应从移植后28 d内启动，持续至移植后100 d[2]。对于高危人群，特别是急慢性GVHD患者，可考虑适当延长疗程或重新启动CMV激活预防，应用至免疫抑制剂减量[49]–[50]。

建议：①对于CMV IgG阴性患者，优先选用CMV血清学阴性或去白细胞的血制品；②对于CMV IgG阳性患者可在高危人群中使用来特莫韦预防，移植后28 d内启动，持续至移植后100 d，也可根据患者免疫重建状况酌情调整预防疗程。

六、CMV病毒血症的治疗

为预防CMV病发生而实施的针对CMV病毒血症的治疗，称为抢先治疗。

（一）一线治疗

更昔洛韦和膦甲酸钠均为一线药物，后者适用于血细胞减少的患者[51]。缬更昔洛韦也可考虑作为一线治疗的选择，治疗有效率为56.0％～91.4％，但不适合用于口服吸收困难的患者[52]–[54]。

（二）二线治疗

难治性CMV血症的治疗称为二线治疗，可选择一线方案中未使用的抗CMV药物进行单药或联合治疗，也可加用细胞毒性T细胞。研究显示加用细胞毒性T细胞（包括CMV特异性T细胞）治疗有效率可达60％～90％[55]–[57]。我国相关研究显示，CMV特异性T细胞治疗4、6周CMV转阴率分别为76.8％、89.5％[58]。静脉注射免疫球蛋白用于难治性CMV感染的疗效尚存争议[3]。

（三）治疗启动时机

一旦检出CMV血症阳性，原则上需积极干预，但治疗启动阈值在各中心之间差异较大[59]。近年来发现部分CMV病患者前期并无CMV血症或诊断时不伴CMV血症，这给CMV治疗启动阈值的设定提出了挑战[26]。临床工作中可结合患者具体情况选择治疗启动时机，如一周内连续2次检测阳性或单次检测高拷贝水平。

（四）治疗停药时机

我国多数中心采用连续2次CMV-DNA检测阴性或连续2次检测均低于抢先治疗启动阈值作为停药指标[11],[18],[26],[60]。在欧洲，这一标准也得到了多数中心和相关指南的推荐，并规定2次检测应至少间隔3～4 d[4],[59]。基于我国临床诊疗的现状，建议连续2次检测（每周2次）CMV-DNA均为阴性或低于抢先治疗阈值作为停药标准。

建议：①更昔洛韦或膦甲酸钠可作为抢先治疗的一线药物，缬更昔洛韦也可考虑用于一线治疗；②二线治疗可选择一线未使用的抗CMV药物进行单药或联合治疗，可加用CMV特异性T细胞；③一旦检出CMV血症阳性，原则上需积极干预，启动抢先治疗的阈值可根据患者具体情况和各单位所采用的检测方法进行调整；④连续2次检测（每周2次的频率）CMV-DNA阴性或低于抢先治疗阈值作为抢先治疗的停药标准。

七、CMV病的治疗

（一）CMV肺炎

更昔洛韦单药治疗作为一线方案，膦甲酸钠作为更昔洛韦不耐受时的替代，也有学者采用更昔洛韦联合膦甲酸钠作为二线治疗方案[1],[36],[61]–[62]。加用静脉注射免疫球蛋白和糖皮质激素是否能够带来获益仍然存在争议[61]。

（二）CMV视网膜炎

通常采用玻璃体内注射更昔洛韦联合全身抗CMV治疗的策略[63]。建议邀请有经验的眼科医师进行评估及局部治疗。

（三）CMV脑炎

CMV脑炎最常采用更昔洛韦联合膦甲酸钠治疗，其次为更昔洛韦或膦甲酸钠单药治疗[64]。由于现有抗CMV药物在脑脊液中的浓度仅为血清的24％～67％，且更昔洛韦与膦甲酸钠在体外具有一定的协同作用，因此联合治疗可能是更佳选择[64]–[65]。也有研究者采用CMV特异性T细胞成功治疗难治性CMV脑炎[65]–[66]。

（四）其他类型CMV病

针对其他类型CMV病的研究较少，可参照CMV肺炎的建议进行治疗。

建议：①CMV肺炎：更昔洛韦作为CMV肺炎的一线治疗选择，膦甲酸钠作为替代，更昔洛韦联合膦甲酸钠作为二线方案；②CMV视网膜炎：玻璃体内注射更昔洛韦联合全身抗CMV治疗为一线方案；③CMV脑炎：更昔洛韦联合膦甲酸钠作为治疗CMV脑炎的一线选择，难治性CMV脑炎患者可加用CMV特异性T细胞。

八、结语

CMV感染是严重影响造血干细胞移植患者预后的重要合并症。本共识的发表应有助于提高国内CMV感染的诊疗水平，进而改善该类患者的预后。随着新药研发以及临床研究的深入，未来将会有更多的治疗方法问世并推动本共识的持续更新。
